# RGB Gait Recognition Using Large Vision Models for Industrial Access Control

**DOI:** 10.3390/jimaging12090458

**Published:** 2026-09-20

**Authors:** Jiaqi Bai, Huijuan Wu, Yan Liu, Jinjiang Qin, Xiaoying Li

**Affiliations:** 1 School of Electronic Information Engineering, Inner Mongolia University, Hohhot 010021, China; 32456076@mail.imu.edu.cn (J.B.); 32456061@mail.imu.edu.cn (Y.L.); 32556065@mail.imu.edu.cn (J.Q.); 32456031@mail.imu.edu.cn (X.L.); 2 Inner Mongolia Key Laboratory of Intelligent Communication and Sensing and Signal Processing, Inner Mongolia University, Hohhot 010021, China

**Keywords:** industrial access control, RGB gait recognition, large vision models, background suppression, cross-view recognition

## Abstract

Identity verification is important for safety management at industrial access points. Gait recognition provides contactless identity cues as workers pass through a gate, but public datasets rarely combine card-swiping, moving barriers, standard workwear, safety helmets, directional occlusion, and changing illumination. In this application-oriented empirical study, we evaluated 1500 RGB access sequences from 150 workers in our previously introduced industrial gait dataset. Each worker was recorded in five passage sequences in each of two opposing, slightly elevated views. Using the grouped, memory-efficient BiggerGait* framework, we compared DINOv2 ViT-S/14, DINOv3 ViT-S/16, and DINOv3 ViT-S+/16 under the same downstream configuration. The human-prior branch did not consistently produce stable person-centered masks when trained from scratch and transferred poorly between backbones. We therefore evaluated raw RGB, background-suppressed RGB, and background-suppressed RGB with a one-patch boundary expansion. Strict suppression mainly improved same-view recognition, whereas the expanded input increased mean cross-view Rank-1 for all three backbones in the present evaluation. This pattern is consistent with a benefit from reducing scene interference, while limited boundary context may recover useful body-edge cues for matching opposing views. DINOv3-S+ with the expanded input achieved same-view and cross-view mean Rank-1 accuracies of 96.3% and 88.3%, respectively.

## 1. Introduction

Reliable identity verification is important for safety management at industrial sites. Card-based access systems are widely used, but cards may be lost, shared, or used by someone other than the registered holder. Fingerprint recognition requires deliberate contact and may interrupt passage at busy entrances. Face recognition can also be affected by safety helmets, masks, downward head poses during card-swiping, and changes in illumination. An additional contactless biometric cue is therefore useful for verifying identity during normal passage.

Gait recognition identifies individuals from body shape and walking dynamics without requiring active cooperation. Existing methods mainly use silhouettes, skeletal keypoints, or RGB sequences. Silhouettes depend on accurate person segmentation, while skeletal keypoints may become unreliable under occlusion, rear views, and incomplete body observations. These limitations are particularly relevant in industrial access-control settings, where card-swiping, moving barriers, safety helmets, standard workwear, and partial out-of-frame regions affect both segmentation and pose estimation. RGB sequences preserve body appearance, contour, and local motion cues, but they also retain fixed equipment and other scene-specific information that is unrelated to identity and may reduce robustness across passage directions or acquisition conditions.

Recent RGB gait methods have shown that frozen large vision models (LVMs) can extract informative person-related cues directly from RGB sequences. BigGait introduced a framework based on frozen DINOv2 features [1,2], while BiggerGait extended this direction by exploiting complementary information from multiple transformer layers [3]. However, the reliability of these methods in industrial access-control settings remains insufficiently understood. Fixed equipment, reflective surfaces, card-swiping, moving barriers, standard workwear, and illumination changes may introduce scene-specific correlations or obscure useful body cues. Reliable deployment therefore requires gait representations that preserve appearance, boundary, and motion information while remaining robust to scene variation across passage directions and operating conditions.

To investigate these issues, we use the original RGB recordings from the industrial access-control dataset introduced in our previous study [4], comprising 1500 passage sequences from 150 workers at an operational checkpoint. Each worker completed five passages in each of two opposing, slightly elevated views over a period of more than one month. The recordings capture card-swiping, occlusion caused by the moving barrier, safety helmets, standard workwear, reflective gate surfaces, and illumination changes. Within the grouped BiggerGait* framework, we compare DINOv2 ViT-S/14, DINOv3 ViT-S/16, and DINOv3 ViT-S+/16, hereafter denoted DINOv2-S, DINOv3-S, and DINOv3-S+, respectively. DINOv2-S serves as the reference backbone, DINOv3-S represents the standard small-scale DINOv3 architecture, and DINOv3-S+ adopts the higher-capacity ViT-S+ architecture with a SwiGLU feed-forward network [5]. The input resolutions are adjusted so that all three backbones produce the same 32×16 spatial token grid and use an identical downstream gait model. We further evaluate a backbone-independent foreground-processing strategy based on person segmentation. Raw RGB, background-suppressed RGB, and background-suppressed RGB with one-patch boundary expansion are evaluated for each backbone, allowing recognition performance to be compared across backbone and input choices under a common downstream configuration.

The contribution is an application-oriented empirical assessment using established components: the grouped BiggerGait* architecture, frozen DINO backbones, and YOLOv8 segmentation. We do not introduce a new gait-recognition architecture or a new dataset. The study examines their behavior and computational trade-offs in an operational industrial setting.

The empirical contributions are summarized as follows:

1. We establish an RGB evaluation on our previously introduced industrial gait dataset, using 1500 sequences from 150 workers to examine recognition under bidirectional views, gate-specific actions, and occlusion.
2. We compare DINOv2-S, DINOv3-S, and DINOv3-S+ using a common 32×16 token grid and the same downstream configuration, reporting recognition accuracy together with measured inference costs.
3. We analyze human-prior transfer and compare raw RGB, background-suppressed RGB, and background-suppressed RGB with one-patch boundary expansion for same-view and cross-view recognition.

## 2. Related Work

### 2.1. Gait Representations and Robust Recognition

Silhouettes are widely used in gait recognition because they remove most color, texture, and background information. Early work represented a walking sequence using the gait energy image [6]. Subsequent deep methods improved representation learning by treating silhouettes as unordered sets, modeling local body motion, or combining global and local features [7,8,9]. DeepGaitV2 and OpenGait further established strong architectures and standardized evaluation settings [10,11]. More recent methods address imperfect observations, including noisy silhouettes, incomplete sequences, and unconstrained tracklets [12,13,14].

Structural representations describe gait using body keypoints, skeletal heatmaps, parsing maps, or human meshes. PoseGait, GaitGraph, and GaitGraph2 model temporal relationships between body joints [15,16,17], while SkeletonGait converts pose estimates into image-like skeletal representations [18]. HMRGait incorporates three-dimensional body shape and pose, and ParsingGait uses fine-grained human parsing maps [19,20]. Multimodal methods further combine complementary representations [21,22]. Although these approaches reduce scene interference, their performance depends on the quality of segmentation, pose estimation, parsing, or mesh recovery. This dependence is important in industrial access-control settings, where helmets, standard workwear, card-swiping, moving barriers, elevated cameras, and partial body truncation can produce unreliable foreground or structural observations.

For IMU-based zero-shot activity recognition, TEZARNet uses videos as auxiliary information [23], while SEZ-HARN provides skeleton-video explanations for its predictions [24]. In gait recognition, our previous study evaluated DeepGaitV2, SkeletonGait, and SkeletonGait++ on the industrial dataset and introduced Denoising Progressive Compression (DPC) and Segment-wise Quality Aggregation (SQA) to improve occlusion robustness using silhouette and skeleton representations [4]. Using the same dataset, the present study examines RGB gait recognition with frozen DINO backbones and the grouped BiggerGait* framework, focusing on backbone selection, background suppression, and boundary-context retention.

### 2.2. RGB-Based Gait Recognition

RGB sequences retain body appearance, contours, internal structure, and local motion without requiring gait recognition to rely entirely on a predefined silhouette or pose representation. Their additional information can be valuable when segmentation or keypoint estimation is unreliable, but it also introduces clothing texture, illumination changes, and scene-specific cues. GaitEdge takes RGB frames as input and learns a constrained, silhouette-like intermediate representation with trainable body boundaries [25]. AttenGait uses attention to identify informative regions from rich visual modalities, including optical flow [26], while DenoisingGait combines human priors, diffusion features, and geometric matching to reduce gait-irrelevant information in walking videos [27].

Large vision models have recently provided stronger pretrained representations for RGB gait recognition. BigGait transforms frozen DINOv2 features through human-prior, appearance-transformation, and denoising branches [2]. BiggerGait further exploits complementary information from multiple intermediate layers [3]. GaitMax combines semantic features from large vision models with part-level kinematic modeling [28]. These studies show that pretrained visual features can capture rich appearance, structural, and motion information from RGB sequences. In industrial access-control environments, however, RGB models may also learn identity-irrelevant correlations from fixed equipment, reflections, illumination changes, and direction-dependent background or occlusion. Reliable deployment therefore requires gait representations that preserve discriminative person-related information while remaining robust to scene-specific variation.

### 2.3. Gait Datasets and Industrial Access-Control Scenarios

CASIA-B supports multiview evaluation under normal walking, clothing changes, and carried objects [29], while OU-MVLP extends evaluation to a much larger population and range of viewpoints [30]. GREW and Gait3D contain more diverse subjects, cameras, backgrounds, and walking conditions collected in less constrained environments [31,32]. CCPG focuses on clothing variation [33], whereas SUSTech1K provides synchronized camera and LiDAR observations under changes in viewpoint, clothing, carrying, occlusion, and illumination [34].

Although these datasets cover controlled, unconstrained, clothing-change, and multimodal conditions, they do not specifically represent routine industrial access-control passage. Such scenarios combine card-swiping, moving barriers, safety equipment, standard workwear, elevated cameras, direction-dependent occlusion, and repeated passages over time. Data collected in an operational industrial environment are therefore necessary to assess whether RGB gait recognition remains reliable under these application-specific conditions.

## 3. Materials and Methods

### 3.1. Industrial Access-Control Dataset and Input Construction

#### 3.1.1. Acquisition Setting and Protocol

The data were collected at an operational industrial access-control checkpoint as workers entered and exited the site. Each passage involved card swiping, entering the access lane, and walking through the gate. Data collection spanned more than one month and covered different dates and times of day. Workers wore standard workwear and safety helmets. Card swiping introduced arm extension, downward head poses, and brief pauses, while the moving barrier caused time-varying partial occlusion as workers passed through the gate.

Two RGB cameras were installed at opposite ends of the access passage, one in front of the gate and the other behind it. Both cameras were mounted slightly above head height and angled downward. The 000 view captured workers walking toward the camera, whereas the 180 view captured them walking away from it. The two views differed in visible body regions, card-swiping actions, and occlusion patterns.

**Figure 1:**
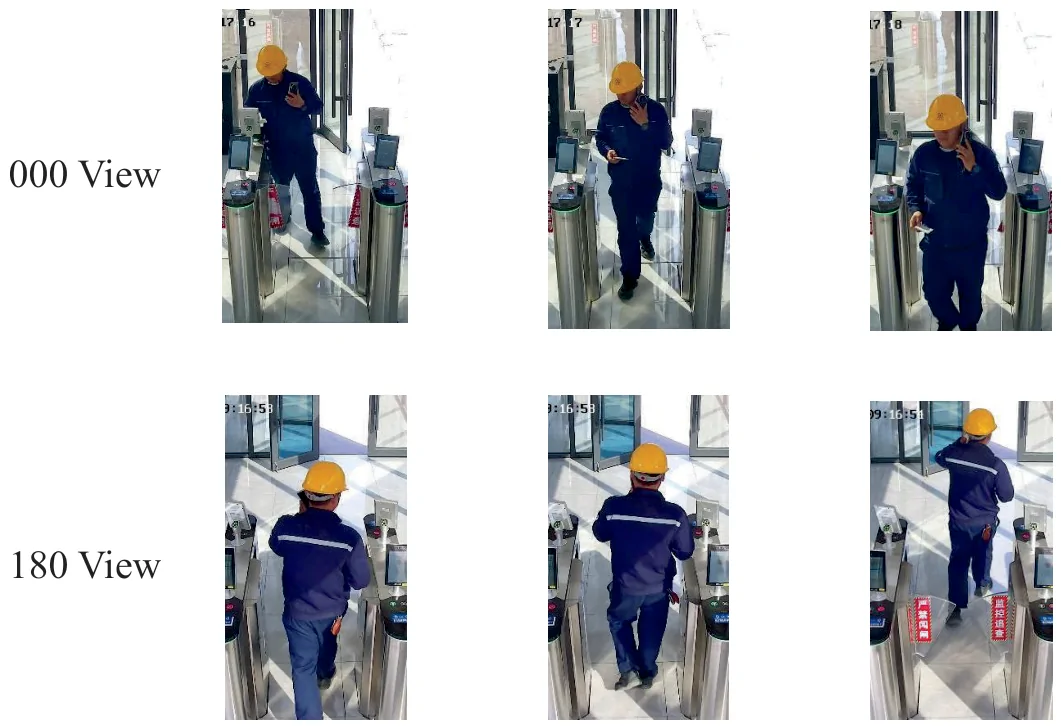
Representative frames from the industrial access-control dataset. The 000 view shows workers approaching the camera, whereas the 180 view shows workers moving away from it.

The dataset, previously introduced for industrial gait recognition [4], comprises 150 workers, each with five sequences in each direction, yielding 1500 RGB videos. The present study focuses on the original RGB sequences, which preserve appearance, body-contour, and scene information that is not fully represented by silhouettes or keypoints. Representative frames are shown in Figure 1.

Each source video contained approximately 60 frames. The central 45 frames were retained in temporal order to reduce incomplete body crops near the beginning and end of a passage. During training, 36 frames were sampled from each sequence and kept in chronological order, whereas all 45 frames were used for testing. This procedure preserves the temporal progression of the passage and its changing occlusion while reducing redundant frames and standardizing sequence length.

#### 3.1.2. Human-Prior Branch

Following BigGait, the human-prior branch of BiggerGait obtains a human mask from high-level LVM features without requiring pixel-level supervision [2,3]. Let ${\left\{f_{i}\right\}}_{i=1}^{N}$ denote the intermediate feature maps extracted by the frozen LVM backbone. To train or adapt the human-prior branch, the highest-level feature map $f_{N}$ is processed by a lightweight auto-encoder:

(1) $$p={\mathrm{softmax}}_{c}\;\left(E(f_{N})\right),\;{\hat{f}}_{N}=D\left(p\right),\;L_{\mathrm{rec}}=\frac{1}{C_{N}H_{N}W_{N}}{∥f_{N}-{\hat{f}}_{N}∥}_{F}^{2},$$

where *E* and *D* are point-wise linear layers, equivalent to 1×1 convolutions in the spatial representation. The encoder produces a two-channel probability map p, and the decoder restores the original feature dimension. The softmax operation is performed along the channel dimension.

The two-channel probability map is converted into a binary human mask using the foreground-selection and thresholding procedure adopted from BigGait:

(2) $$m=S_{\tau }\left(p\right),\;\tau =0.5,$$

where $S_{\tau }\left(\cdot \right)$ denotes foreground selection and binarization. After the intermediate features are grouped and projected as described in Section 3.2.1, the mask is resized and applied to each group representation:

(3) $$g_{j}^{m}=R_{j}\left(m\right)⊙g_{j},\;j\in \left\{1,2,\ldots ,J\right\},$$

where $g_{j}$ is the projected feature of the *j*th layer group, $R_{j}\left(\cdot \right)$ resizes the mask to the corresponding spatial resolution, and ⊙ denotes element-wise multiplication. The mask is broadcast along the channel dimension. This mask is the only explicitly introduced human prior in BiggerGait.

**Figure 2:**
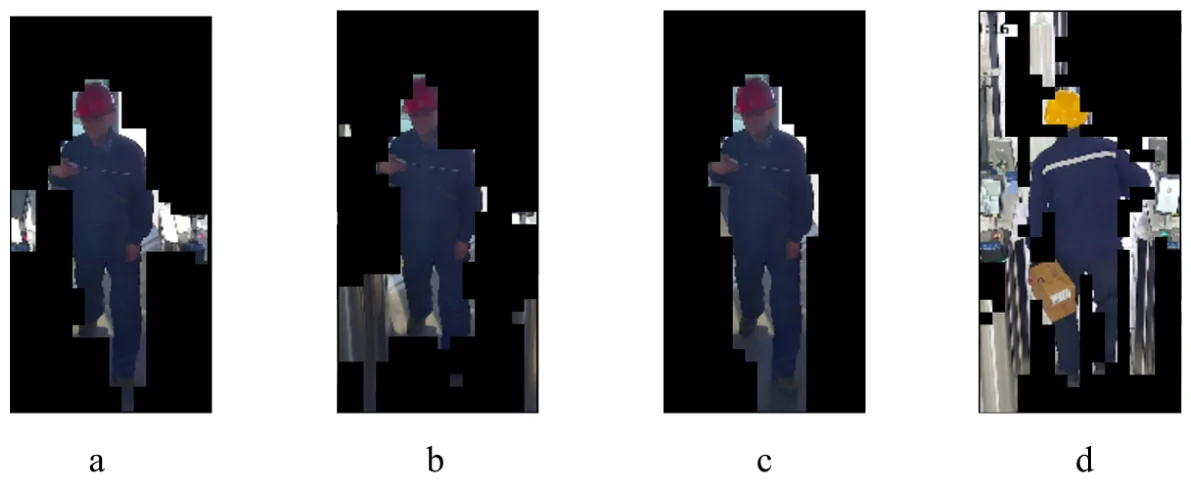
Representative human-prior outputs: (**a**) the released weights with DINOv2-S; (**b**) direct transfer of the released weights to DINOv3-S; (**c**) adaptation of the released weights to DINOv3-S; and (**d**) a representative DINOv3-S+ result obtained by training the human-prior branch from scratch.

For each backbone, a separate human-prior branch was randomly initialized and trained using only the 90 training identities, while the LVM backbone remained frozen. The resulting masks were sometimes inconsistent across frames and extended into reflective regions of the gate. Figure 2d shows a representative DINOv3-S+ result; similar cases were observed for DINOv2-S and DINOv3-S.

We also examined the transferability of the mask-branch weights released by the BiggerGait authors. Figure 2a shows the released weights with DINOv2-S, Figure 2b shows their direct transfer to DINOv3-S, and Figure 2c shows the result after adaptation using the 90 training identities. Although the weights could also be loaded into DINOv3-S+, they did not produce a stable person-centered mask. These observations suggest that the human-prior response depends on the backbone feature space, motivating the backbone-independent foreground processing described in the following section.

#### 3.1.3. Backbone-Independent Foreground Processing

As a backbone-independent alternative to the learned human-prior branch, we construct foreground-aware RGB inputs using explicit person masks. The masks are estimated in image space before LVM feature extraction, allowing the same preprocessing procedure to be applied to all three backbones.

Frames were processed at 256×128 pixels. Person masks were generated using YOLOv8-seg [35], fine-tuned on approximately 1800 annotated frames from the same checkpoint containing only the 90 gait-training identities. The 60 gait-test identities were excluded, although segmentation-training and gait-test data were collected during overlapping periods. The segmentation model was trained separately and kept fixed for offline preprocessing.

Using these masks, we evaluated three input variants: raw RGB, background-suppressed RGB, and expanded background-suppressed RGB. Let $I\in R^{3\times 256\times 128}$ denote an RGB frame and $M\in {\left\{0,1\right\}}^{256\times 128}$ its binary person mask. The three inputs are defined as

(4) $$\begin{array}{cc} I_{\mathrm{raw}}\; & =I,\\ I_{\sup}\; & =I⊙M,\\ I_{\exp}\; & =I⊙\left(M⊕K_{17}\right), \end{array}$$

where ⊙ denotes element-wise multiplication, with the mask broadcast across the three RGB channels; ⊕ denotes morphological dilation; and $K_{17}$ is a 17×17 square structuring element. One dilation iteration expands the mask by up to 8 pixels along each image axis. Pixels outside the corresponding mask are set to zero before backbone normalization.

**Figure 3:**
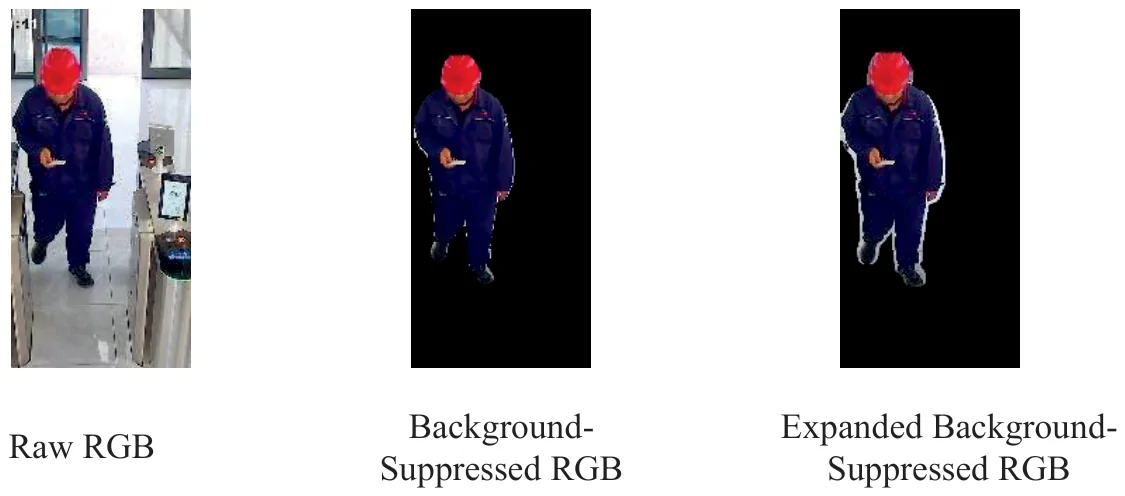
Examples of the three RGB input variants: raw RGB, background-suppressed RGB, and background-suppressed RGB with one-patch expansion.

Strict background suppression removes most fixed scene content, but segmentation errors may exclude body-boundary regions such as parts of helmets, shoulders, or shoes. The expanded mask retains a narrow region around the estimated person boundary. The 8-pixel expansion at the 256×128 processing resolution was selected to correspond to one backbone patch after resizing. DINOv2-S resizes each frame to 448×224, giving a scale factor of 1.75 and mapping the expansion to 14 pixels, which matches its 14×14 patch size. DINOv3-S and DINOv3-S+ resize each frame to 512×256, giving a scale factor of 2 and mapping the expansion to 16 pixels, which matches their 16×16 patch size. The expanded input therefore retains approximately one patch of context beyond the estimated person boundary while preserving the common 32×16 spatial token grid. Examples of the three input variants are shown in Figure 3. This patch-aligned setting was evaluated as a common design choice; its optimality across expansion widths, viewpoints, or operating environments has not been established.

### 3.2. Industrial RGB Gait Recognition with BiggerGait*

#### 3.2.1. Grouped Layer-Wise Gait Representation

**Figure 4:**
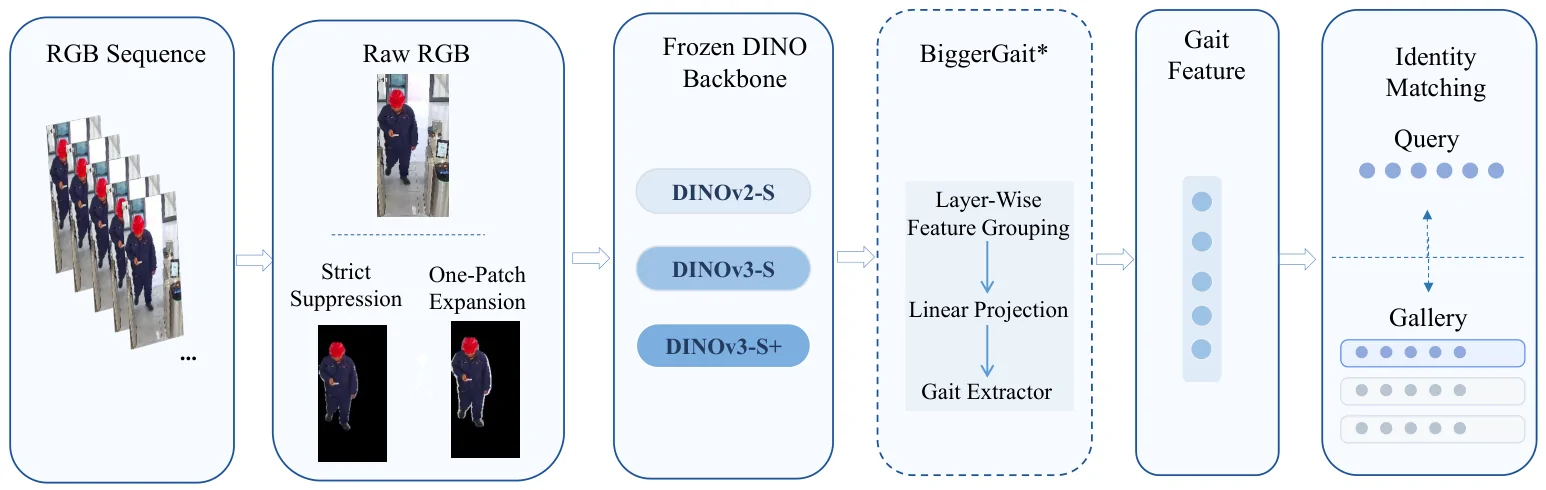
Overview of the grouped BiggerGait* framework. Three RGB input variants are processed by one of the frozen DINO backbones. The layer-wise features are grouped, projected, and encoded into a gait feature for query–gallery matching.

We used the grouped, memory-efficient BiggerGait* framework proposed by the BiggerGait authors [3]. The overall pipeline is illustrated in Figure 4. Standard BiggerGait assigns a separate projection and gait encoder to every LVM layer. Although this preserves complementary layer information, it requires substantial memory and computation for 12 independent gait heads. Because the experiments were conducted using a single GPU, we adopted the authors’ grouping strategy.

For each RGB frame, a frozen DINO backbone outputs feature maps from 12 intermediate transformer layers. The class token and, where present, the register tokens, are removed. The remaining patch tokens from each layer are reshaped into a two-dimensional feature map. In experiments without the human-prior branch, the backbone receives raw RGB, background-suppressed RGB, or expanded background-suppressed RGB.

The 12 intermediate feature maps are divided by depth into J=6 consecutive groups, with two adjacent layer feature maps concatenated along the channel dimension in each group. Each concatenated group feature is processed by a group-specific lightweight projection that reduces its output to 16 channels. Bilinear interpolation then aligns all projected group representations to a common spatial resolution. In experiments with the human-prior branch, the resized human mask is applied to each projected group representation. The resulting masked or unmasked group representations are subsequently processed by the gait extractors.

This grouping strategy reduces the number of gait-head forward passes from 12 to 6. The six depth groups share P=2 gait encoders: one for the shallow groups and one for the deep groups. Each group produces a gait representation, and the resulting distances are fused at test time. We followed the recommended J=6 and P=2 configuration. The DINOv2 and DINOv3 backbones remained frozen throughout gait training.

#### 3.2.2. DINO Backbones and Token Alignment

We compared DINOv2-S [1], DINOv3-S, and DINOv3-S+ [5], with their configurations summarized in Table 1. A common 32×16 spatial token grid was selected to preserve the 2:1 aspect ratio of the input crops and keep the spatial resolution entering the downstream gait modules consistent. DINOv2-S processes 448×224 images with 14×14 patches, while the DINOv3 backbones process 512×256 images with 16×16 patches. Both settings yield 512 spatial tokens per frame after excluding class and register tokens where present. This alignment allows the same grouping, projection, and gait-extraction modules to be used across backbones.

All backbones remained frozen during training, so their parameter differences do not change the number of trainable downstream parameters. However, the comparison is token-grid matched rather than strictly compute-matched: DINOv3 processes more input pixels, and DINOv3-S+ uses a larger FFN. Freezing avoids backbone gradient computation but does not eliminate forward inference costs.

#### 3.2.3. Evaluation Protocol

We used closed-set identification with disjoint training and test identities. Of the 150 workers, 90 were assigned to training and 60 to testing. Only the 60 test identities were used to form the gallery and query sets. For each identity, five sequences were available in each of the 000 and 180 views. The first three sequences in each view formed the gallery, and the last two formed the query set.

Four protocols were evaluated, 000→000, 180→180, 000→180, and 180→000, where the left side denotes the gallery view and the right side denotes the query view. The first two protocols measure same-view recognition; the last two measure cross-view recognition across the opposing, slightly elevated directions. The model outputs one representation for each sequence. Query-to-gallery candidates are ranked by Euclidean distance. Rank-1 is the primary identification metric, measuring whether the top-ranked gallery match has the correct identity. Rank-5 is a supplementary retrieval measure indicating whether a correct match appears among the first five ranked candidates; it is not an acceptance criterion for access control. Neither metric measures threshold-based verification performance, which would require separate false-acceptance and false-rejection evaluation. Same-view and cross-view averages are the arithmetic means of the corresponding two protocols. Each protocol contains 120 query sequences. Mean Rank-1 values and percentage-point differences are calculated before rounding to one decimal place.

#### 3.2.4. Training and Inference Configuration

The downstream modules were trained using SGD with an initial learning rate of 0.01, momentum of 0.9, weight decay of 0.0005, and a stepwise learning-rate schedule. Each batch contained four identities and three sequences per identity, with 36 ordered frames per sequence. Triplet loss (margin 0.2) and cross-entropy loss (logit scale 16) were equally weighted. Training used random horizontal flipping, synchronized batch normalization, and a fixed schedule, with the DINO backbones frozen. Both training and inference used FP16. Inference measurements used one 45-frame sequence per batch. After 20 warm-up passes, latency was measured over 100 synchronized forwards and reported as mean ± sample standard deviation; throughput was measured over three windows of 100 forwards. FLOPs and memory were profiled separately: FLOPs covered major convolution, matrix-multiplication, and attention operations, counting one multiply–add as two FLOPs, while memory denotes the peak allocated memory measured using PyTorch 2.7.1.-allocated GPU memory. Measurements covered the recognition model from prepared GPU inputs to output embeddings, excluding preprocessing, data transfer, gallery matching, network communication, and sequence acquisition.

## 4. Results

Our previous study [4] evaluated DeepGaitV2 as a silhouette-based baseline and SkeletonGait and SkeletonGait++ as skeleton-based baselines on the same industrial dataset, together with DPC and SQA experiments. These published evaluations provide the conventional baseline context. The two studies use the same identity partition but different training sequence lengths, so they are not a strictly training-matched comparison. For an established RGB reference within the present experiments, Table 2 includes grouped BiggerGait* with DINOv2-S and the human-prior weights released by its authors, yielding 93.3% same-view and 86.3% cross-view mean Rank-1. Table 3 then compares backbone and input variants within that established framework. Under the respective experimental settings, the RGB approach evaluated here achieved higher recognition accuracy than the silhouette- and skeleton-based baselines reported in our previous study [4], demonstrating its potential for gait recognition on this industrial dataset.

### 4.1. Transfer of Fixed Human-Prior Weights

We evaluated the compatibility of fixed human-prior weights with different backbones. Three configurations were compared: the weights released by the BiggerGait authors with DINOv2-S, their direct transfer to DINOv3-S, and the industrial-adapted weights with DINOv3-S. The LVM backbone and human-prior branch were frozen in all configurations, and only the downstream gait model was trained. The adapted weights were prepared beforehand using only the 90 training identities and remained fixed in this experiment. No test identity or test sequence was used. DINOv3-S+ was not included in the quantitative transfer comparison because neither direct weight transfer nor training from scratch produced a stable person-centered human-prior response under the present setting.

With the released weights, DINOv2-S achieved mean Rank-1 accuracies of 93.3% for same-view recognition and 86.3% for cross-view recognition. Direct transfer to DINOv3-S reduced these results to 91.7% and 78.3%, respectively. Its cross-view accuracy was 5.0 percentage points lower than the 83.3% obtained by DINOv3-S using raw RGB without the human-prior branch in Table 3. Although the released weights could be loaded into DINOv3-S, they did not transfer effectively to its feature space.

Using the industrial-adapted weights increased the same-view and cross-view mean Rank-1 accuracies to 94.6% and 87.9%, respectively. Relative to direct transfer, these results represent improvements of 2.9 and 9.6 percentage points, respectively. Rank-5 accuracy remained high across all configurations, suggesting that the human-prior weights primarily affected top-rank ordering. These results demonstrate that parameter compatibility alone does not ensure effective transfer across backbone feature spaces.

### 4.2. Backbone and Input Construction

We compared the three visual backbones and three input constructions without the human-prior branch. The downstream BiggerGait* structure, training configuration, and evaluation protocol were fixed across all nine combinations. Results are reported in Table 3.

With raw RGB, the same-view mean Rank-1 accuracies were 89.6%, 88.3%, and 88.8% for DINOv2-S, DINOv3-S, and DINOv3-S+, respectively. The corresponding cross-view results were 83.8%, 83.3%, and 86.7%. Although DINOv3-S+ was slightly less accurate than DINOv2-S in the same-view protocols, it improved cross-view mean Rank-1 by 2.9 and 3.3 percentage points over DINOv2-S and DINOv3-S, respectively. These comparisons describe the observed performance under the current evaluation setting.

Strict background suppression increased same-view mean Rank-1 to 94.6%, 94.2%, and 96.7% for DINOv2-S, DINOv3-S, and DINOv3-S+, corresponding to gains of 5.0, 5.8, and 7.9 percentage points over raw RGB. Its effect on cross-view recognition was smaller: accuracy decreased by 0.4 points for DINOv2-S and DINOv3-S+, but increased by 1.3 points for DINOv3-S. Strict suppression therefore primarily benefited same-view recognition.

Adding one patch of boundary context produced same-view mean Rank-1 accuracies of 93.8%, 93.8%, and 96.3%, and cross-view accuracies of 84.6%, 86.3%, and 88.3%, respectively. Relative to strict suppression, same-view accuracy decreased by 0.8, 0.4, and 0.4 percentage points, whereas cross-view accuracy increased by approximately 1.3, 1.7, and 2.1 points. Thus, expansion increased the two-direction cross-view mean for all three backbones, with small reductions in the same-view mean. These gains did not occur in every individual cross-view protocol. For DINOv2-S, expansion reduced 000→180 Rank-1 from 81.7% to 80.0% but increased 180→000 from 85.0% to 89.2%. For DINOv3-S, both directions improved, from 83.3% to 85.8% and from 85.8% to 86.7%, respectively. For DINOv3-S+, 000→180 decreased from 85.8% to 85.0%, whereas 180→000 increased from 86.7% to 91.7%. Thus, the mean gains for DINOv2-S and DINOv3-S+ were driven by the 180→000 direction, while DINOv3-S benefited in both directions.

Overall, strict suppression produced the highest same-view accuracy, while one-patch expansion provided a better balance between the two protocol types. DINOv3-S+ with expanded input achieved 96.3% same-view and 88.3% cross-view mean Rank-1. The latter was the highest cross-view result among the nine combinations, while its same-view accuracy remained close to the overall best result.

Across three independent training repetitions of DINOv3-S+ for each input construction on the same identity split, same-view Rank-1 was 89.17±0.42%, 96.81±0.24%, and 96.25±0.00% for raw RGB, suppressed, and expanded inputs, respectively (mean ± sample standard deviation). The corresponding cross-view values were 86.67±0.42%, 86.11±0.24%, and 87.78±0.48%. These repeated-run means preserve the pattern of higher same-view accuracy with strict suppression and higher cross-view accuracy with expansion. The three-run estimates describe observed training variability on this fixed split.

### 4.3. Computational Efficiency

Table 4 reports the inference costs of the complete recognition models, including their frozen backbones. All three configurations have the same 20.612 million trainable downstream parameters.

DINOv2-S had the lowest latency and peak memory and the highest throughput. Compared with DINOv2-S, DINOv3-S required only 0.5% more counted FLOPs but 25.6% higher latency, indicating that similar arithmetic counts do not imply similar execution time. DINOv3-S+ increased FLOPs by 12.8% and latency by 38.6%. Freezing the backbones avoids their backward computation but does not eliminate forward inference costs. These measurements describe computational differences under a common token grid rather than an equal computational budget.

### 4.4. Background Suppression and Deployment Implications

**Figure 5:**
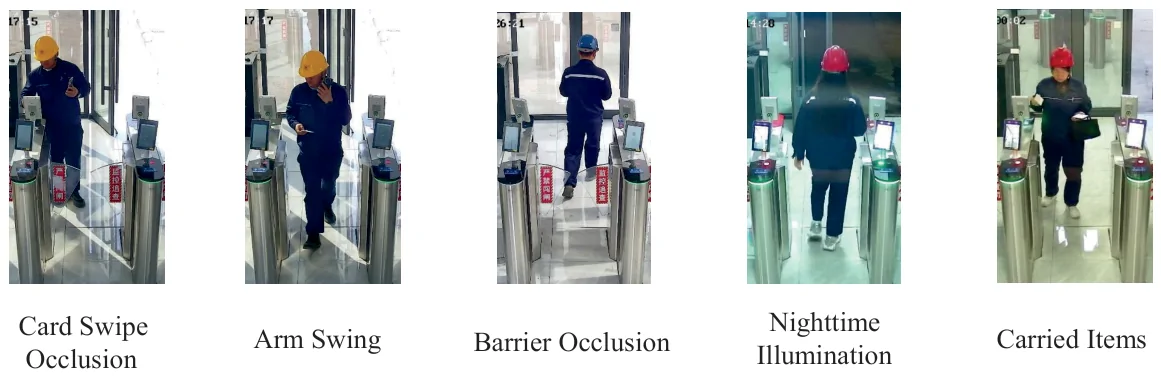
Representative conditions in the industrial access-control passage scenario, including card-swiping, arm motion, barrier occlusion, nighttime illumination, and carried objects.

Industrial passage conditions introduce interference close to the walking person. As illustrated in Figure 5, card-swiping and arm motion change the body contour, while barriers and carried objects cause partial occlusion. Reflective gate surfaces and nighttime illumination further alter the scene appearance. Background processing must therefore suppress stable gate structures without consistently removing helmets, arms, shoes, or other informative regions near the body boundary.

The human-prior results demonstrate the difficulty of learning a stable foreground representation directly from backbone features. Directly transferring the released DINOv2-S prior to DINOv3-S yielded mean Rank-1 accuracies of 91.7% for same-view recognition and 78.3% for cross-view recognition. After adaptation, these values increased to 94.6% and 87.9%, respectively. The human-prior branch should therefore be revalidated and, where feasible, adapted whenever the backbone or application domain changes.

In contrast, YOLOv8-based background suppression operates in the image space and can be shared across different gait backbones. Strict suppression was associated with higher same-view mean Rank-1 in the present evaluation. Expanding the person mask by one patch retained limited boundary context and increased cross-view mean Rank-1 by 1.3, 1.7, and 2.1 percentage points for DINOv2-S, DINOv3-S, and DINOv3-S+, respectively. DINOv3-S+ with the expanded input achieved 96.3% same-view and 88.3% cross-view mean Rank-1. These results suggest a useful trade-off between scene suppression and retention of boundary information in the evaluated setting.

**Figure 6:**
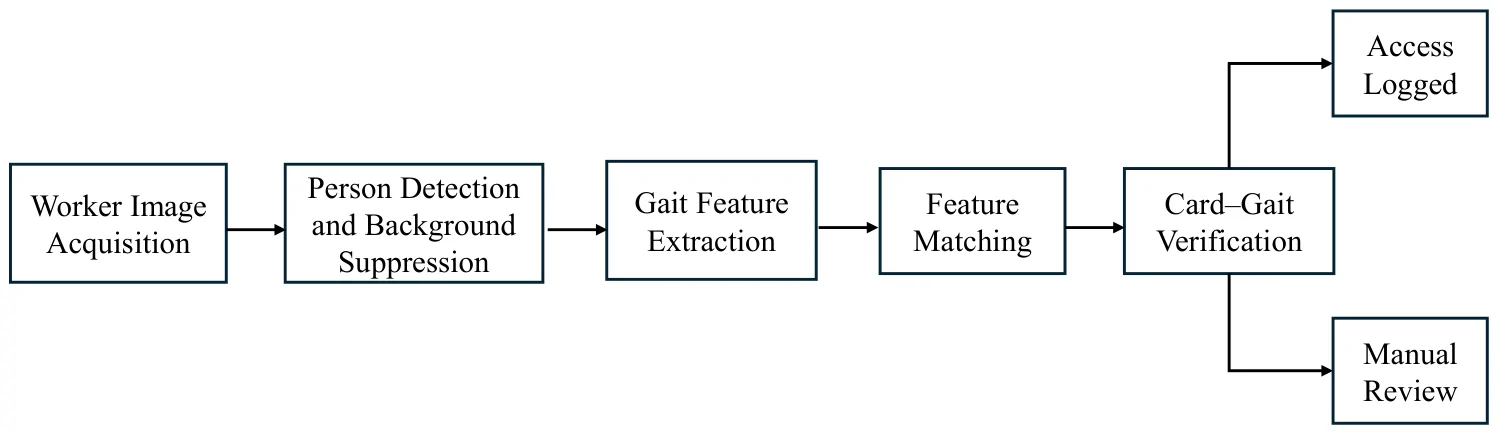
Conceptual workflow for using RGB gait recognition as a secondary identity check in industrial access control.

Figure 6 presents the intended system-level workflow. Worker images are first processed by the segmentation module to detect the person and suppress the background. The processed sequence is then encoded by the frozen DINO backbone and the grouped BiggerGait* model. The resulting gait feature is matched against the registered feature database, and the retrieved identity is compared with the presented access card. Consistent results are recorded as normal passages, whereas mismatches are referred for manual review.

Separating background suppression from gait feature extraction allows the segmentation model to be adapted to workwear, helmets, barriers, and camera conditions without modifying the gait backbone. This modular design also avoids relearning a feature-space-dependent human prior whenever the backbone is replaced. The current evaluation used offline preprocessing, and Figure 6 represents a conceptual workflow rather than a validated real-time implementation. Table 4 measures model inference on a workstation GPU and does not validate performance on representative edge hardware or the intended edge–server system. Future evaluation on representative edge hardware or the intended edge–server system should measure segmentation and preprocessing, 45-frame gait feature extraction, gallery matching, and any network transmission and queuing.

## 5. Discussion

Under the same token grid and BiggerGait* configuration, DINOv3-S+ achieved the highest observed cross-view mean Rank-1 in this dataset, whereas DINOv3-S did not consistently outperform DINOv2-S. A newer backbone therefore does not necessarily provide better gait recognition; performance also depends on model capacity and input construction. The inference measurements in Table 4 additionally show that DINOv2-S is more efficient on the tested GPU, whereas DINOv3-S+ trades additional inference cost for higher observed cross-view accuracy.

The human-prior branch transferred poorly from DINOv2-S to DINOv3-S and required adaptation to recover its performance. In contrast, YOLOv8-based background suppression operates directly on RGB frames and can be shared across backbones. Its main advantage is modularity rather than a consistent accuracy advantage over an adapted human prior.

Strict background suppression mainly improved same-view recognition. One plausible explanation is that removing equipment and reflections reduces irrelevant variation while the visible side of the person remains comparable between gallery and query. Across opposing views, differences in visible body regions, body projection, and barrier occlusion remain after background removal. For DINOv3-S+ with expanded input, the single-run same-view and cross-view means are 96.3% and 88.3%, respectively, a gap of about eight percentage points. The three-run means, 96.25% and 87.78%, show the same limitation. Thus, improved foreground selection does not resolve cross-view matching.

Retaining one patch around the estimated person boundary increased mean cross-view Rank-1 for all three backbones. Expansion may retain visible helmet, shoulder, or shoe pixels omitted by the mask and preserve local contour information, but it also retains nearby scene content. These possibilities cannot be separated by the raw, suppressed, and expanded comparisons alone. The asymmetric cross-view results may reflect differences in card-swiping, visible body regions, barrier occlusion, and illumination.

### Application Scope and Limitations

Public datasets represent different conditions from industrial access control. CASIA-B and OU-MVLP mainly contain multiview walking in relatively open settings [29,30], whereas GREW, Gait3D, and SUSTech1K include more varied backgrounds, illumination, or occlusion [31,32,34]. However, they do not reproduce the same combination of card interaction, moving barriers, reflective equipment, safety workwear, and constrained bidirectional passage. The present results therefore demonstrate the value of background suppression in this industrial setting, but do not establish its effectiveness in open, lightly occluded, or non-industrial scenes.

The evaluated pipeline is intended as a secondary check between the recognized gait identity and the presented access card, with mismatches referred for manual review. For the expanded DINOv3-S+ configuration, 98.3% cross-view Rank-5 accompanies 88.3% Rank-1 in the single-run comparison. A correct identity appearing in a shortlist does not ensure a correct top-ranked decision, and no verification operating threshold or false-acceptance/false-rejection performance has been established. These results therefore do not validate standalone access authorization.

All videos were collected at one checkpoint, with 60 test identities and a single train–test split. The 1500 sequences are repeated observations of 150 workers, not 1500 independent identity samples. The dataset provides focused coverage of industrial passage conditions rather than large-population evidence. Although training and test identities were disjoint, they shared the same site and camera configuration. The results therefore support a controlled within-site comparison but do not establish generalization to other sites, camera heights, viewing angles, distances, or barrier layouts. Different worker populations, workwear, and passage habits may also change recognition performance.

Background suppression remains sensitive to segmentation errors. Expansion may retain body-boundary pixels omitted by the mask, but can also include nearby background and cannot recover fully occluded regions. Its effectiveness depends on mask quality, viewpoint, and person scale, so a one-patch margin is not necessarily optimal in other settings. Further controlled comparisons are needed to distinguish the effects of boundary recovery and background retention.

## 6. Conclusions

This empirical study assessed established RGB gait-recognition components using the original 1500 bidirectional passage videos from 150 workers in our previously introduced industrial access-control dataset. The data include card-swiping, moving barriers, helmets, standard workwear, and illumination changes across times of day. Three DINO backbones were compared with a common grouped BiggerGait* configuration. Because the human-prior branch was strongly tied to its backbone feature space, we also evaluated YOLO-based background suppression as a modular input transformation. Under the present evaluation setting, strict suppression mainly improved same-view recognition, while retaining one patch of context around the person increased mean cross-view Rank-1 for all three backbones. The expanded input can be reused across gait backbones and adjusted separately for site-specific workwear and camera conditions, making it a candidate for further field testing.

## Figures and Tables

**Table 1:** Visual backbones and token-alignment settings. Parameter counts refer to the loaded backbone modules.

Setting	Backbone	Params. (M)	FFN	Input	Patch	Token Grid
DINOv2-S	ViT-S/14	22.057	MLP	448×224	14×14	32×16
DINOv3-S	ViT-S/16	21.597	MLP	512×256	16×16	32×16
DINOv3-S+	ViT-S+/16	28.693	SwiGLU	512×256	16×16	32×16

**Table 2:** Recognition results with fixed human-prior weights (%).

Backbone	Prior Weights	Same-View R1			Cross-View R1			Mean R5	
		000→000	180→180	Avg.	000→180	180→000	Avg.	Same	Cross
DINOv2-S	Released weights	95.0	91.7	93.3	87.5	85.0	86.3	**99.6**	97.9
DINOv3-S	Direct transfer	90.8	92.5	91.7	74.2	82.5	78.3	98.8	98.3
DINOv3-S	Adapted weights	95.8	93.3	**94.6**	87.5	88.3	**87.9**	99.2	**98.8**

*Note:* Bold values indicate the best results in the corresponding summary columns.

**Table 3:** Single-run recognition results for different backbone and input combinations (%). Three-run DINOv3-S+ summaries are reported in the accompanying text.

Backbone	Input	Same-View R1			Cross-View R1			Mean R5	
		000→000	180→180	Avg.	000→180	180→000	Avg.	Same	Cross
DINOv2-S	Raw RGB	90.8	88.3	89.6	80.8	86.7	83.8	98.3	97.5
	Suppressed	95.8	93.3	94.6	81.7	85.0	83.3	**100.0**	96.3
	Expanded	94.2	93.3	93.8	80.0	89.2	84.6	99.6	97.9
DINOv3-S	Raw RGB	89.2	87.5	88.3	81.7	85.0	83.3	98.8	96.7
	Suppressed	95.8	92.5	94.2	83.3	85.8	84.6	99.6	97.1
	Expanded	95.0	92.5	93.8	85.8	86.7	86.3	99.6	98.8
DINOv3-S+	Raw RGB	90.0	87.5	88.8	85.0	88.3	86.7	99.2	97.9
	Suppressed	97.5	95.8	**96.7**	85.8	86.7	86.3	**100.0**	**99.2**
	Expanded	96.7	95.8	96.3	85.0	91.7	**88.3**	99.6	98.3

*Note:* Bold values indicate the best results in the corresponding summary columns.

**Table 4:** Complete-model inference costs without the human-prior branch, measured using FP16, batch size one, and 45 frames per sequence. Latency is mean ± sample standard deviation over 100 forwards; memory denotes peak PyTorch allocation.

Backbone	Total Params. (M)	GFLOPs/Seq.	Latency (ms/Seq.)	Memory (MiB)	Throughput (Seq./s)
DINOv2-S	42.668	2674.228	37.546±0.088	1556.879	26.604
DINOv3-S	42.208	2688.474	47.174±0.065	2047.062	21.109
DINOv3-S+	49.304	3017.808	52.037±0.118	2073.632	19.154

## Data Availability

De-identified silhouette and keypoint representations derived from the same industrial gait dataset are publicly available for non-commercial academic research at https://github.com/baijq233/Industrial-Gait (accessed on 13 September 2026). The original RGB videos contain identifiable appearance and biometric information and are therefore not publicly available. They may be obtained from the corresponding author upon reasonable request, subject to institutional approval and applicable privacy requirements.
